# Topical Application of bFGF Alone for the Regeneration of Chronic Tympanic Membrane Perforations: A Preliminary Case Series

**DOI:** 10.1155/2021/5583046

**Published:** 2021-05-15

**Authors:** Zihan Lou, Zhengcai Lou, Kangfeng Jin, Junzhi Sun, Zhengnong Chen

**Affiliations:** ^1^Department of Otolaryngology-Head and Neck Surgery, Shanghai Jiao Tong University Affiliated Sixth People's Hospital, Shanghai 200233, China; ^2^Otolaryngology Institute of Shanghai Jiao Tong University, Shanghai 200233, China; ^3^Department of Otorhinolaryngology, Yiwu Central Hospital, Yiwu City, 322000 Zhejiang Province, China

## Abstract

**Results:**

A total of 29 patients consisting 13 in the bFGF alone group and 16 in the myringoplasty group were finally included in the analysis. Of the 13 patients in the bFGF alone group, the perforations were small in 6 and medium in 7; the etiology was secondary to COM in 11 and to trauma in 2. One patient with an unhealed perforation continued bFGF treatment until 6 months, while the others stopped at 3 months. Of the seven medium-sized perforations, none of the five COM perforations closed, while the two traumatic perforations achieved complete closure within 2 and 4 weeks, respectively. The successful closure rate was 28.6% (2/7). Successful closure was achieved in 66.7% (4/6) of the six small perforations with COM, with a mean closure time of 4.75 weeks. Of the 16 patients in the myringoplasty group, all perforations were medium-sized and were secondary to COM in 15 cases and traumatic in 1 case; all achieved complete closure.

**Conclusions:**

bFGF alone facilitated the repair of chronic traumatic perforations and small perforations with COM, but not medium-sized perforations with COM. These observations indicated that the regenerative conditions of traumatic perforations are better than those of COM perforations when using bFGF alone, and that graft materials could play a critical role in the regeneration of larger-sized chronic perforations with COM.

## 1. Introduction

Chronic tympanic membrane (TM) perforation is common and usually requires surgical repair using underlay, overlay, or underlay–overlay techniques. Common graft materials are cartilage, perichondrium, temporalis fascia, and fat. Some biological materials are used to repair chronic perforations [1–5]. The regenerative effects of basic fibroblast growth factor (bFGF) on the TM are striking. bFGF is produced in situ after TM laceration and facilitates healing of perforations by stimulating the proliferation and differentiation of endothelial cells, fibroblasts, keratinocytes, and neovascularization at the margins [6]. In clinical studies, bFGF alone or combined with a gelatin sponge accelerated eardrum healing and improved the closure rate of traumatic perforations compared with spontaneous healing [7–11]. Recently, bFGF has been used to repair chronic perforations with encouraging results [12–18]. However, all of these studies also included biological materials, such as atelocollagen, silicone [12, 13, 15, 17], gelatin sponges, or fibrin glue [14, 16, 18]. Biological materials alone can repair chronic perforations [1–5]. Thus, the addition of biological materials may have affected the effects of bFGF on the repair of chronic perforations. There have been no previous reports on the topical application of bFGF alone for the repair of chronic perforations; so, it remains unclear whether bFGF alone can facilitate the regeneration of chronic perforations. Therefore, this study was performed to evaluate the effects of topical bFGF alone in the repair of chronic TM perforations.

## 2. Materials and Methods

### 2.1. Study Design

This prospective case-control study was approved by the Human Research Ethics Committee of Yiwu Central Hospital, Yiwu, China, as guided by local policy, national laws, and the World Medical Association Declaration of Helsinki. Informed consent was obtained from all patients.

### 2.2. Patients

The study recruited 36 patients with chronic TM perforations from the Yiwu Central Hospital Otology Clinic. The inclusion criteria were age > 20 years; chronic TM perforation with chronic otitis media (COM) or trauma for >18 months; dry perforation for >3 months and no epithelial invagination or cholesteatoma mass; central, small (<1/8 of TM area), or medium (1/8–1/4 of the TM area) perforation; sufficient air in the mastoid antrum and tympanic cavity on temporal bone computed tomography (CT) and no abnormal soft tissue shadow or abnormalities in the auditory ossicles or their linkages on CT and endoscopy; and willing to undergo bFGF eardrop treatment or surgical treatment. Patients with active otitis media, a history of ear surgery, a large (>1/4 of the TM area) or marginal perforation, involvement of the malleus, and chronic otorrhea were excluded. Pure-tone audiometry (PTA) was performed before and at 3 months after treatment or perforation closure at the standard frequencies (air conduction thresholds) of 0.5, 1, 2, and 3 kHz. When the threshold at 3 kHz was missing, it was interpolated by averaging the thresholds at 2 and 4 kHz. Age, sex, size and position (anterosuperior, anteroinferior, posterosuperior, and posteroinferior), duration of perforation, etiology, myringosclerosis, and pre- and posttreatment PTA were recorded. The patients were divided into two groups, i.e., bFGF alone and myringoplasty with autologous perichondrium graft groups.

### 2.3. Surgical Procedures

#### 2.3.1. bFGF Alone Group

All treatments were performed in the otology outpatient clinic. An inoffice myringoplasty was performed after taking a pretreatment photograph of the TM. The external auditory canal (EAC) was cleaned with a cotton swab soaked in povidone-iodine solution. After topical application of 4% lidocaine jelly to the surface of the TM for 15 minutes, the epithelium was removed circumferentially around the edge of the perforation using a delicate right-angled hook by endoscopy to create a fresh wound surface. Approximately, 0.1–0.15 mL (2–3 drops) of recombinant bovine bFGF solution (21,000 IU/5 mL; Yi Sheng, Zhuhai, Guangdong, China) was applied to the TM along the EAC; no scaffolding material was used [9–11]. To keep the perforation edges moist, the patient self-administered bFGF drops twice a day. In the side-lying position, the patient gently pulled the auricle upward to straighten the EAC and instilled the bFGF solution; the perforated ear was kept upward for at least 30 minutes.

#### 2.3.2. Myringoplasty Group

A 4 mm diameter 0° rigid endoscope (18 cm in length) and high-definition monitor were employed in all cases. All patients were operated on under general anesthesia. A 1 cm long skin incision was created on the medial side of the ipsilateral tragus, and tragal perichondrium was harvested. The perforation edges were de-epithelialized and refreshed. Myringosclerotic TM remnants were preserved if present. Then, the perichondrium graft was trimmed; it was 1–2 mm larger than the freshened perforation edges. The tympanic cavity was tightly filled with Gelfoam soaked in antibiotic ointment to the level of the perforation; this supported the perichondrium graft. The perichondrium graft was placed underneath the TM remnant and annulus. Gelfoam was used to splint the graft laterally up to the level of the isthmus. The EAC was packed with gauze soaked in erythromycin ointment up to the tragus incision, which was not sutured.

### 2.4. Follow-Up and Outcome Evaluation

In the bFGF alone group, follow-up was scheduled once weekly for 4 weeks after the initial hospital visit and then every 2 weeks until complete closure of the perforation or up to 3 months. The TM surface needed to be moist, and the TM was examined repeatedly by endoscopy at all follow-up visits. Clinical events, such as a change in perforation size, TM closure, and purulent otorrhea, were photographed in color. All patients were treated for 3 months, and then any patient with an unhealed perforation was offered the choice of continued bFGF treatment, abandoning treatment, or surgical myringoplasty. In the myringoplasty group, the packing gauze and Gelfoam were removed from the EAC at 14 days after surgery to allow the grafts to be visualized endoscopically. Audiometric evaluation was carried out, and perforation closure was evaluated endoscopically at the end of postoperative month 3 in both groups.

## 3. Results

### 3.1. Patient Profiles

The 36 patients initially enrolled in the study were divided into bFGF alone (17 patients) and endoscopic myringoplasty (19 patients) groups, as stated above. However, four patients in the bFGF alone group and three in the endoscopic myringoplasty group were subsequently excluded due to failure to attend follow-up for 3 months. Therefore, a total of 29 patients, including 13 in the bFGF alone group and 16 in the myringoplasty group, were finally included in the study.

The 13 patients in the bFGF alone group (Table 1) had a mean age of 48.5 ± 5.1 years (range: 37–61 years) and consisted of seven women and six men with nine right and four left perforations. The perforation was small in six cases and medium-sized in seven. The perforation was secondary to COM in 11 patients and traumatic in 2. Of the two patients with traumatic perforation, the perforation had been present for 18 months in one and 3 years in the other. The perforation position was anterosuperior in 1 patient and anteroinferior in 12.

The 16 patients in the endoscopic myringoplasty group had a mean age of 47.3 ± 6.4 years (range: 42–68 years) and consisted of 11 women and 5 men with nine right and seven left perforations. All perforations were medium-sized and were secondary to COM in 15 cases and traumatic in 1 case. The perforation position was anterosuperior in 3 patients and anteroinferior in 13.

### 3.2. Endoscope Observation

**Figure 1:**
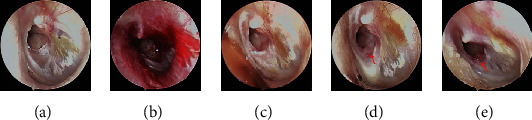
(Patient 1): a 37-year-old woman with COM and a left medium-sized perforation. Pretreatment perforation (a), freshened edges (b), and edges after 2 (c), 7 (d), and 12 (e) weeks of treatment. Red arrows indicate a moderate granulation reaction.

**Figure 2:**
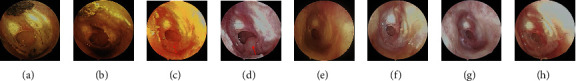
(Patient 2): a 54-year-old woman with COM and a left medium-sized perforation. Pretreatment perforation (a) and the perforation after 2 (b), 6 (c), 8 (d), 12 (e), 18 (f), 19 (g), and 23 (h) weeks of treatment. Red arrows indicate moderate granulation reaction.

**Figure 3:**
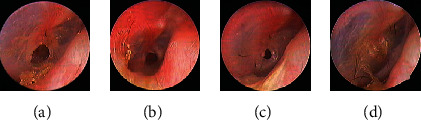
(Patient 8): a 39-year-old man with a right traumatic medium-sized perforation. The 1-week pretreatment perforation (a) and the perforation after 18 months (b) and 3 (c) and 4 (d) weeks of treatment.

**Figure 4:**
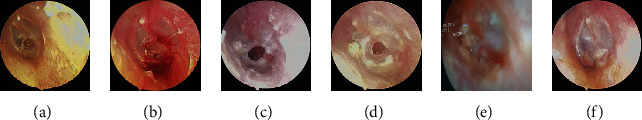
(Patient 9): a 55-year-old man with a left traumatic medium-sized perforation. The perforation at 3 years before treatment (a), freshened edges (b), and the perforation at 3 days (c) and 1 (d), 2 (e), and 8 (f) weeks after starting treatment.

Of the 13 patients in the bFGF alone group, only 1 (patient 2) with an unhealed perforation requested continued treatment with bFGF until 6 months and then underwent endoscopic transtympanic cartilage–perichondrium myringoplasty; the others with unhealed perforations abandoned treatment at 3 months. Of the seven medium-sized perforations, two were traumatic (patients 8 and 9), and five were secondary to COM. Of the latter, none achieved closure: the size of the perforation did not change significantly in three cases (patients 1 [Figure 1], 3, and 13), but decreased by 20% in patient 2 (Figure 2) and 10% in patient 7. Surprisingly, both traumatic perforations achieved complete closure in 4 [patient 8 (Figure 3)] and 2 [patient 9 (Figure 4)] weeks. Closure was successful in 28.6% (2/7) of cases, with a mean closure time of 3 weeks.

**Figure 5:**
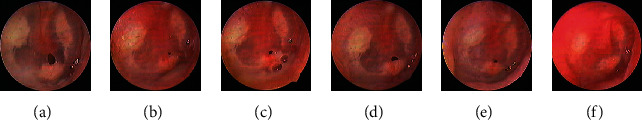
(Patient 12): a 48-year-old woman with COM and a right small perforation. Pretreatment perforation (a) and the perforation at 3 (b), 4 (c), 5 (d), 6 (e), and 8 (f) weeks after starting treatment.

Of the six small perforations with COM, four achieved complete closure [patients 5, 6, 10, and 12 (Figure 5)] in 4, 4, 3, and 8 weeks, respectively. The size of the perforation was unchanged in patient 4 and decreased by 30% in patient 11. Successful closure was achieved in 66.7% (4/6) of cases, with a mean closure time of 4.75 weeks. Morphologically, a moderate granulation reaction was seen at the inferior edge of the perforation in three medium-sized perforations (Figures 1 and 2), while a slight inflammatory reaction and thickened edges were found in three small perforations and one medium-sized perforation. No significant changes occurred in three small and three medium-sized perforations.

**Figure 6:**
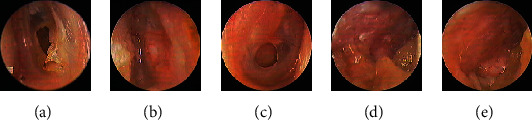
Underlay myringoplasty with perichondrium graft. Preoperative perforation (a) and the perforation at 2 weeks postoperatively (b) in a 33-year-old woman with a 2-year history of a traumatic perforation. Preoperative perforation (c) and the perforation at 3 weeks (d) and 4 weeks (e) postoperatively in a 51-year-old woman with COM.

Of the 16 patients with medium-sized perforations in the endoscopic myringoplasty group, 15 with COM and one with trauma achieved complete closure at 3 months postoperatively, representing successful closure rate of 100% (16/16) (Figure 6).

## 4. Hearing Outcomes

In the bFGF treatment group, of the seven patients with hearing improvement, one patient with a small perforation that showed no change in size had an improvement of 6.25 dB in PTA, while four patients with complete closure of their perforations had a mean improvement of 11.4 dB, and two patients with medium-sized perforations had a mean improvement of 5 dB but also a mean decrease of 15% in perforation size. Of the six patients with no change in PTA, two had complete closure of small perforations, one showed a 30% decrease in the size of the perforation, and the remaining three had medium-sized perforations that showed no change in size (Table 1). In the endoscopic myringoplasty group, 16 patients with complete closure of their perforations had a mean improvement of 12.7 dB in PTA.

## 5. Discussion

Some clinical studies have shown encouraging results for bFGF treatment of chronic perforations [12–18]. However, they all used additional biological materials. Although these materials released bFGF steadily, the materials themselves can repair chronic perforations [1–5]. A reliable study design should exclude known confounding factors. Clinical and experimental studies have demonstrated that bFGF is not ototoxic, and that short-term bFGF application does not lead to middle ear cholesteatoma [6, 17, 19, 20]. In addition, clinical and experimental studies showed that bFGF alone facilitated the regeneration of traumatic and chronic experimental perforations [6, 8, 10, 11, 19]. However, whether bFGF alone facilitated the regeneration of human chronic perforation was unclear. The present study was performed to determine whether bFGF alone facilitated the regeneration of human chronic perforations. At physiological pH and temperature, the in vitro half-life of fibroblast growth factor-2 activity is approximately 12 hours [21]. Therefore, twice-daily topical application of bFGF was reasonable in this study.

Of the seven medium-sized chronic perforations in the bFGF alone group, none of those with COM showed closure, while both chronic traumatic perforations achieved complete closure. This implies that bFGF promotes the repair of chronic traumatic perforations, but not chronic perforations with COM. This also indicates that the regenerative conditions of traumatic perforations are better than those of the perforations with COM. We speculated that they have different pathological mechanisms. The failed healing associated with chronic traumatic perforation is due to extensive epithelialization and abnormal migration of epithelium at the edges [22, 23], but no changes in the collagen structure of the fibrous layer in the TM [24]. In contrast, recurrent middle ear inflammation and bacterial toxins may inhibit endogenous healing in chronic perforations with COM. Some authors have reported that higher concentrations of matrix metalloprotease (MMP) proenzymes in chronic wound beds degrade the wound matrix necessary for optimal healing and result in reduced cellular mitogenic activity and decreased growth factor levels in the residual tissue, affecting angiogenesis and epithelial proliferation that are prerequisites for wound healing [25, 26]. Other authors found that chronic perforation with COM involved longstanding structural changes in the fibrous layer with a disorganized collagen layer in the TM [24]. Demidova-Rice et al. [27] found that the signaling pathways that initiate cellular and tissue responses after injury may be impeded during healing of chronic wounds. Nevertheless, bFGF mainly stimulates the proliferation of fibroblasts and revascularization of the fibrous layer in the TM remnant, thereby facilitating TM healing [6, 12]; it does not change the disorganized collagen structure in chronic perforations with COM. In addition, 15 medium-sized perforations with COM achieved complete closure in our myringoplasty group. The results indicated a critical role of graft material in the regeneration of larger chronic perforations with COM.

We observed a significant inflammatory reaction and edema at the edges of the two medium-sized chronic traumatic perforations. This implies that the signaling pathways that initiate cellular and tissue responses may have been normal in the remnant TM. We speculate that topical application of bFGF initiated the inflammatory reaction of TM healing and restored the normal TM healing process. The two chronic traumatic perforations healed within 2 and 4 weeks. In addition, the epithelialization and abnormal migration of epithelium at the edges of traumatic perforations can be inhibited or reversed by a moist environment [24, 28]. Although some experimental studies have implied that direct application of bFGF alone improved the healing of experimental chronic TM perforations [19], the experimental chronic perforation was similar to a chronic traumatic perforation. In an experimental study, a model of chronic TM perforation was defined as a perforation persisting at 6–8 weeks that failed to close [29]. Nevertheless, some studies have suggested that TM perforation resulting from the edge of recreated microflaps is not an ideal model of chronic TM perforation, and there is still no rat model of chronic tympanic perforation, only models of delayed healing [30, 31]. In addition, although 66.7% (4/6) of small perforations with COM achieved complete closure, the mean closure time of 3 weeks for the two traumatic medium-sized perforations was shorter than the 4.75 weeks for the four small perforations with COM, also implying that the pathological mechanisms of chronic traumatic and COM perforations differ.

The results presented here indicate that bFGF repair of chronic perforations with COM was unsatisfactory. Although 66.7% (4/6) of small perforations with COM achieved complete closure, we cannot be sure that this resulted from the actions of bFGF. Some studies have implied that a moist environment facilitates proliferation of granulation tissue at the edges and aids eardrum healing [25, 28]. Santos et al. [32] found that the difference in the effective closure rate was not significant between FGF-2 and sterile water groups (40% vs. 57%, respectively). Similarly, topical epidermal growth factor (EGF) alone improved the closure rate of traumatic and chronic traumatic perforations [33, 34], but the effect was disappointing for chronic perforations with COM. Ramsay et al. [35] divided 17 chronic perforations into EGF (*n* = 8) and placebo groups (*n* = 9); after 2.6 months, complete perforation closure was observed in only one ear in the placebo group and in none in the EGF group. Although bFGF improved the closure rate and shortened the closure time compared with spontaneous healing, the same institution subsequently found that bFGF did not significantly improve the healing outcome compared with 0.3% ofloxacin eardrops or EGF alone [36–38]. Although Ramsay et al. [35] inferred that the absence of a desired effect was due to the lack of stripping of the edges to enable EGF to repair chronic perforations with COM and suggested that stripping the edge might have improved the healing results, in other studies, EGF or bFGF has resulted in high healing rates for chronic traumatic perforations, without edge stripping [33, 39].

Why was there a marked disparity between ours and other studies? We did not apply any biological material to seal the perforation; we applied bFGF eardrops only, while the other studies simultaneously applied biological materials to seal the perforations [12–18]. These biological materials not only stimulate the perforation margin to induce the inflammatory reaction and epidermal proliferation but also replace the disorganized collagen layer of chronic perforations, guiding epithelial migration and thereby closing the perforation [32, 40]. Nevertheless, our study was better in evaluating the effects of bFGF on repairing perforations with COM, as twice-daily eardrops maintained the activity of bFGF. Unfortunately, topical eardrops do not diffuse evenly to all perforation edges, especially the superior edges of medium- or large-sized perforations. In two cases, proliferation of granulation tissue at the inferior edges was seen, while there was no reaction at the superior edges. We speculate that bFGF does not change the endogenous healing mechanism, but induces only proliferation of target cells and angiogenesis at perforation edges. All but one of the perforations was anteroinferior. While not opposing application of bFGF to repair chronic perforations with COM, in this study, topical application of bFGF alone did not achieve the desired effect. When bFGF did heal chronic perforations with COM, it required a long time, which would limit its broad clinical application. In comparison, endoscopic transtympanic underlay myringoplasty with perichondrium grafts would benefit most patients and is a short operation that does not require an additional incision.

The limitations of this study included the small sample size, single-center design, and lack of a control group. Moreover, although previous clinical studies showed that the use of bFGF alone for repairing traumatic perforations did not cause reperforation or acquired cholesteatoma in the long-term [20], clinical studies of bFGF combined with biological scaffold obtained similar findings [13–17]. As the follow-up period was short in this study (only 3 months), it remains unclear whether the use of bFGF alone for repairing chronic perforations will result in long-term reperforation and acquired cholesteatoma. In addition, further studies are required to compare rimming of the perforation combined with bFGF with rimming of the perforation alone to determine the biological efficacy of bFGF.

## 6. Conclusions

bFGF alone facilitated the repair of chronic traumatic perforations and small perforations with COM, but not medium-sized perforations with COM. These observations indicated that the regenerative conditions of traumatic perforations are better than those of COM perforations when using bFGF alone, and that graft materials could play a critical role in the regeneration of larger chronic perforations with COM.

## Figures and Tables

**Table 1:** Patient profiles.

	Side	Age, years	Sex	Duration, years	Size	Position	Etiology	Myringosclerosis	History of otorrhoea?	Pretreatment PTA (dBHL)	Posttreatment PTA (dBHL)	Follow-up	Successful?	Decrease of size
Patient 1	L	37	F	11	Medium	Anterosuperior	COM	No	Yes	27.25	27.25	3 months	No	Unchanged
Patient 2	L	54	F	31	Medium	Anteroinferior	COM	Yes	Yes	30	22.5	6 months	No	Decrease 20%
Patient 3	R	48	F	19	Medium	Anteroinferior	COM	No	Yes	22.25	22.25	3 months	No	Unchanged
Patient 4	R	61	F	34	Small	Anteroinferior	COM	No	Yes	16.25	16.0	3 months	No	Unchanged
Patient 5	L	54	M	49	Small	Anteroinferior	COM	No	Yes	12.5	12.5	4 weeks	Yes	Closure
Patient 6	R	48	M	8	Small	Anteroinferior	COM	No	Yes	18.25	12.0	4 weeks	Yes	Closure
Patient 7	R	51	M	12	Medium	Anteroinferior	COM	No	Yes	17.5	15.0	3 months	No	Decrease10%
Patient 8	R	39	M	1.5	Medium	Anteroinferior	Trauma	No	No	22.5	12.25	4 weeks	Yes	Closure
Patient 9	L	55	M	3	Medium	Anteroinferior	Trauma	No	No	27.5	10.5	2 weeks	Yes	Closure
Patient 10	R	47	F	9	Small	Anteroinferior	COM	No	Yes	12.5	12.25	3Weeks	Yes	Closure
Patient 11	R	49	M	26	Small	Anteroinferior	COM	No	Yes	16.0	16.0	3 months	No	Decrease30%
Patient 12	R	48	F	13	Small	Anteroinferior	COM	Yes	Yes	17.25	5.0	8 weeks	Yes	Closure
Patient 13	R	39	F	7	Medium	Anteroinferior	COM	No	Yes	20.25	20.5	3 months	No	Unchanged

L: Left; R: Right; F: Female; M: Male; COM: chronic otitis media; PTA: Pure-tone audiometry.

## Data Availability

All data generated or analyzed during this study are included in this published article.

## Additional Points

The English in this document has been checked by at least two professional editors, both native speakers of English. For a certificate, please see http://www.textcheck.com/certificate/jWwfOY.
